# It’s a long, long walk: accessibility to hospitals, maternity and integrated health centers in Niger

**DOI:** 10.1186/1476-072X-11-24

**Published:** 2012-06-27

**Authors:** Justine I Blanford, Supriya Kumar, Wei Luo, Alan M MacEachren

**Affiliations:** 1 GeoVISTA Center, Department of Geography, The Pennsylvania State University, University Park, PA, USA; 2 Department of Epidemiology, Graduate School of Public Health, University of Pittsburgh, Pittsburgh, PA, USA

**Keywords:** Accessibility, Health facilities, Niger, Infectious disease, Measles, Meningitis, Geographic information system, Crisis management, Vaccination, Seasonal variation

## Abstract

**Background:**

Ease of access to health care is of great importance in any country but particularly in countries such as Niger where restricted access can put people at risk of mortality from diseases such as measles, meningitis, polio, pneumonia and malaria. This paper analyzes the physical access of populations to health facilities within Niger with an emphasis on the effect of seasonal conditions and the implications of these conditions in terms of availability of adequate health services, provision of drugs and vaccinations. The majority of the transport within Niger is pedestrian, thus the paper emphasizes access by those walking to facilities for care. Further analysis compared the change in accessibility for vehicular travel since public health workers do travel by vehicle when carrying out vaccination campaigns and related proactive health care activities.

**Results:**

The majority of the roads in Niger are non-paved (90%). Six districts, mainly in the region of Tahoua lack medical facilities. Patient to health facility ratios were best in Agadez with 7000 people served per health facility. During the dry season 39% of the population was within 1-hours walk to a health center, with the percentage decreasing to 24% during the wet season. Further analyses revealed that vaccination rates were strongly correlated with distance. Children living in clusters within 1-hour of a health center had 1.88 times higher odds of complete vaccination by age 1-year compared to children living in clusters further from a health center (p < 0.05). Three key geographic areas were highlighted where access to health centers took greater than 4 h walk during the wet and dry season. Access for more than 730,000 people can be improved in these areas with the addition of 17 health facilities to the current total of 504 during the dry season (260,000 during the wet season).

**Conclusions:**

This study highlights critical areas in Niger where health services/facilities are lacking. A second finding is that population served by health facilities will be severely overestimated if assessments are solely conducted during the dry season. Mapped outputs can be used for future decision making processes and analysis.

## Background

Access to health care is multi-dimensional comprising of several factors that include availability, acceptability, financial accessibility and geographic accessibility [1]. Here, we focus on geographic or physical accessibility to health care in Niger since this has been shown to be an important factor in the use of healthcare [2-10].

Niger is one of the poorest countries. It has the 9^th^ lowest Gross Domestic Product, with the highest birth rate in the world at 51.6 births per 1000 population [11]. With 66% of the population living on less $1/day [12], not only does Niger have a high birth rate but also has the third highest mortality rate in under five year olds (259 per 1000 births) in Africa [13] and high maternal mortality ratio (1600 per 100,000 live births) [13]. In addition, the people of Niger are exposed to a wide variety of diseases that occur both during the wet and dry season. For example, measles, meningitis, polio and pneumonia are prevalent during the dry season (generally between October and March) while Cholera (June to September) and Malaria (August to September) are highest during the wet season [12-19]. Malaria, alongside pneumonia, meningitis and measles, can cause high mortality in children under-five [13,20]. Neonatal mortality is also high: from 2000-2003, 17% of the deaths among children under five years of age were due to neonatal causes [13,20]. Furthermore, DPT3 vaccine uptake rate was only 34.7% by age 12 months among children aged 12–23 months in 2006 [21,22]. These figures illustrate some of the health challenges in Niger.

Eighty percent of Niger is desert and is continually hampered by cycles of drought and desertification. The climate of Niger is hot and dry with maximum temperatures reaching in excess of 40 °C during the summer months. Annual rainfall varies between the regions with the least rain falling in the Northeast (< 100 mm) and the most in the South (~ 500 mm). Most of the rain falls in a two-month period and can cause extensive damage through flooding [23-26]. The harsh climate along with poor infrastructure can make it a challenge to provide medical services. This has been well illustrated with the countrywide “Roll Back Malaria” campaign where a house-to-house approach was used to provide optimal coverage in the distribution of bed nets. Remote villages were accessed using “*25 camels, 45 donkeys, and more than a dozen boats”*[27] and nets distributed to posts within 5 km of each village [28]. Access to health facilities are not only a challenge in Niger (further illustrated through a video by UNICEF during the polio vaccination campaign in 2005 [29]), but also in the surrounding countries such as Burkina Faso [30] and Nigeria [31]. For example, in Nigeria it is not uncommon for women in rural areas to walk 26 miles to seek medical assistance [31], while in Burkina Faso, many of the small health centers—though serving at least 2000 villages-have no doctors and are run by nurses [30]. During outbreaks, these small health centers lack the facilities to deal with an increase in patient numbers, as highlighted during the meningitis outbreak of 2007 [30].

Access to health services can help reduce the impact of diseases such as pneumonia, malaria, measles, meningitis and polio that may be controlled through medicines or vaccinations. For example, a recent study by O’Meara *et al.*[5] found that hospitalization from malaria was greatly reduced when primary health facilities were within a two-hour walk. Another study showed that risk of mortality doubled after a walking distance of four-hours to a health center [6]. In Niger walking 6 h [32] or 14 km to seek treatment is not unusual [33,34]. Similar findings have been documented in other African countries. For example, in Kenya, 40% of the population must travel in excess of an hour to the nearest primary health care facility [4]; 64% of pregnant women spent at least 60 min travelling to a health facility in Ghana mainly by bicycle or walking [35].

Utilization rate of health facilities diminishes with distance [2,7-10] and the quality of transportation and road conditions [36]. With road conditions, particularly those that are dry weather roads, the problems of accessibility can be further exacerbated during the wet season when many roads can become impassable particularly to motor transport [35,37,38]. Few studies have examined the impact of wet and dry seasons on health-seeking behavior. However, the few that have [6,,^ 38^-^40^] show that the wet season can reduce accessibility of health services [38], and that hard-to-reach villages are less likely to seek medical help due to increased costs associated with travel, which are further accentuated during the wet season [39].

Though much is known about the household-level factors that are related to complete and timely vaccination of children [41-53], lack of data on travel times has resulted in few studies carefully examining the impact of travel-time to a health center on childhood vaccine uptake [2]. Using Geographic Information Systems (GIS) to assess utilization of health centers [2,54], determining optimal location of health facilities [3] and modeling access to health facilities [55] is not new. However, in this study we show how vulnerable certain populations are by calculating realistic continuous travel times across different surfaces and illustrating how access to health centers may be affected at different times of the year with seasonal changes by walking and vehicular travel. In areas where populations are most vulnerable we further investigate how physical accessibility may be improved through the placement of additional health facilities.

## Methods

In this section, we provide details on data assembled and the methods used to obtain the results. In the first three sections, we provide an overview of the health care system in Niger, data available (or not) at each level, describe the population data used in the analysis, and sketch the methods used to derive travel time data. Next, we outline the accessibility surface modeling process applied to derive estimates of health care accessibility. Finally we provide a detailed description of the analyses we conducted.

### Health facilities and health care system in Niger

The organization of the health system in Niger is described by Ridde and Diarra [56]. In summary, the health system is organized at three levels within each district: hospitals, integrated health centers (*Centre de santé intégré*, CSI) and health posts [56]. Although there are three levels of health facilities within Niger, digital data on the location of these facilities are available only for the first two. The location of health facilities in Niger is from 2009 and was obtained through the FAO GeoNetwork Portal ( http://www.fao.org/geonetwork). These are from World Health Organisation (WHO) and were originally based on data from Ministry of Health and Ministry of Hydraulics, Niger, 1995. The health facilities dataset provides the location of health facilities but does not contain additional information about the facilities, equipment or their utilization. Included with these data were the location of maternity centers, many of which are located at the same location as hospitals and integrated health centers. Since maternal mortality rates are high in Niger [12], these were also included. Therefore analysis reported here is restricted to hospitals (N = 50) (1 Community, 3 National, 15 Department, and 31 District), integrated health centers (N = 400) and maternity centers (N = 54). Although additional facilities may exist in Niger, no public information is available for these.

### Population data for Niger

Data on the distribution of population settlements throughout Niger was obtained through the FAO GeoNetwork Portal. These data were compiled by the Ministry of the Environment of Niger. Each settlement record contained a latitude and longitude and population data. The data is current from 2005.

### Travel time data for Niger

Euclidean distances can overestimate the population that is within 1-hour of a health facility by 19% therefore using transport network, elevation and other natural barriers can provide more accurate estimates [4] as used here. In Niger, public transportation is virtually non-existent, with bus services running only between the main cities (e.g. Niamey, Agadez, Arlit, Maradi and Zinder [57]). Transport is mainly by foot, but can also include the use of animals such as horse and camel [58]. Many of the secondary roads in Niger are dry weather roads [37], which become impassable after rains. To account for seasonal variations, travel times were assigned to the different road types and are summarized in Table 1. Realistic travel speeds were obtained from a variety of sources during the dry and wet season (see Tables 1, 2, and 3[37,59-66]). The best analog identified by which to estimate reduction in travel speeds during the rainy season is a study that produced validated travel velocity on different road surfaces in Honduras [62,63]; thus, this source was used to derive the estimates of reduced travel speed used here.

**Table 1:** Walking and Vehicular travel times on different road surfaces in Niger during the dry and wet season

**Road Type**	(a) Dry season*		(b) Wet season**	
	**Walking**	**Vehicle**	**Walking**	**Vehicle**
**Primary (Paved)**	5 km/h (12 min/km)	30 km/h (2 min/km)	4 km/h (15 min/km)	20 km/h (3 min/km)
**Secondary & Tertiary**	4 km/h (15 min/km)	17 km/h (3.5 min/km)	3 km/h (20 min/km)	8 km/h (7.5 min/km)
			Impassable where intersect with a river	Impassable where intersect with a river
Track through sandy desert*	2 km/h (30 min/km)	6.78-13.54 km/h (4.4 min/km – 8.6 min/km)	1.7 km/h (40 min/km)	Impassable

**(a) Dry Season:** Documented travelling times in Niger is very limited therefore travelling times along the different types of roads during the dry season were constructed from a variety of sources and are summarized below. **(b) Wet Season:** Documented travelling times in Niger is very limited therefore travelling times along the different types of roads during the rainy season were constructed from a variety of sources and are summarized below.

*traveling from Agadez to Bilma (approximately 650 km) (across the Tenere Desert) walking with and riding camels takes 2 weeks therefore walking speed was estimated at approximately 2 km/h [59] (similar to Tanser et al., [65]); travel using local vehicles takes 2–4 days therefore travel speed was calculated to be 6.78–13.54 km/h [60].

** The desert piste roads (highlighted as track) as well as many of the secondary roads are dirt or gravel and can become impassable after heavy rains [37]. Reduction in travelling speeds are estimates that were based on travel speeds from Nelson [62].

**Table 2:** **Walking speed across different land cover types during the dry and wet season. Travel speed were compiled from Pozzi & Robinson [**[64]**]**

**Land cover type**	**Dry season**	**Wet season**
	**Walking**	**Walking**
**Open or sparse vegetation**		
Includes open grassland, open grassland with shrubs, sparse grassland, croplands (>50%), croplands with woody vegetation	3 km/h (20 min/km)	2 km/h (30 min/km)
**Deciduous Shrub land/woodland**		
Irrigated Croplands	1.5 km/h (40 min/km)	1 km/h (60 min/km)
Closed grassland		
**Desert**	1.5 km/h	1 km/h
*Stony desert*	1.5 km/h	Impassable
*Bare Rock*	1.5 km/h	Impassable
**Water bodies**	Impassable	Impassable
**Cities**	4 km/h (15 min/km)	3 km/h (20 min/km)

**Table 3:** **Effect of slope on travel speed by foot and vehicle. Travel speed were compiled from Pozzi & Robinson, [**[64]**]**

**Slope (%)**	**Walking**	**Vehicle**
**0–2**	100	100
**2–5**	80	80
**5–8**	60	60
**8–12**	50	50
**12–16**	40	40
**16–32**	20	20
**>32**	10	10

### Accessibility surface model

A variety of methodologies have been used to investigate accessibility based on distance and travel time between locations (see [2,55,62,64,67-69]). Cost-distance algorithms are among the most common of these methods. They have been extensively used to calculate accessibility [62,64,67,70,71] for a variety of applications (e.g. town planning [67], analysis of infrastructure for planning of emergency services [62,63], health care planning [7], identification of health catchment areas [4], livestock policy development [64] and identifying global accessibility [72]) and will be used in this study. Since a high proportion of travel in Niger is by foot, and many roads are unpaved and can be impassable in the rainy season, a raster rather than network-based method for estimating cost-distance was chosen. This allows for any route between locations to be considered, not just those along established roads. A raster approach generates a travel “cost” surface that equates distance with the cost to travel through any given cell in the raster grid used to represent the cost surface. Cost can be derived by any relevant metric. Specifically, we used the cost-distance function in ArcGIS 10 Spatial Analyst to calculate the path with the least cost; using travel time as the cost metric, the function will determine the fastest route across a surface. Generating the cost surface is accomplished by calculating the linear and diagonal accumulated least cost of getting to the nearest source (in this case the health centers) from each grid cell [70].

A *friction surface* that represents the characteristics of the landscape (in this case the speed of travel) was used to describe how each surface type will impact walking, the main form of travel in Niger and surrounding countries ( [33,35,39]), and vehicular speed of travel during the dry and rainy season. For example, the speed of travel will be fastest on roads (Tables 1) and non-road travel speed will vary by landscape type (e.g. walking speed will be faster across flat, open grasslands than through sandy deserts or steep rocky slopes) (Tables 2,3). Permanent water bodies will act as a barrier. During the wet season non-permanent/fluctuating water bodies will also act as a barrier. Thus, for this study four *friction surfaces* were created using a road network, land cover, slope and water. Each dataset was rasterized and merged into a single *friction surface*. All data were projected to an equal area projection and the analysis performed at a 1 km grid resolution. A description of each of the datasets is provided next.

#### Roads

Road network data were constructed from a variety of sources. These included digitizing of roads from static maps (Routard.com) and incorporating them with roads from digital data (VMAP0, DCW, FAO-GeoNetwork) as well as verifying primary routes using satellite imagery (GoogleMaps^TM^). Data from the digital sources were checked for connectivity and fixed for dangling nodes and snapped. Each road type was assigned the speed value from Table 1. Roads classified as “Unknown” were analyzed and assigned secondary or ‘track’ based on where they were located. For example, if an unknown road was connected to a secondary road it was reclassified as secondary. Roads located in the northern part of Niger were likely to be in the desert and therefore assigned as track. During the rainy season, speed of travel may be reduced and roads may become impassable due to flooding (as highlighted in several articles [24,25,73-76]). To account for this, secondary and track road types that were found intersecting water bodies were classified as impassable during the rainy season otherwise were assigned a reduced travel speed, as illustrated in Table 1. The procedure outlined assumes an absence of bridges which may not be accurate in all cases, however, data on location of bridges are not available and for these secondary and track roads lack of bridges was considered likely.

Data for land cover, water (comprised of water bodies and streams) and slope were used to create realistic travel times across different surfaces. Further details of each of these datasets are discussed next. ***Land cover:*** The representation of land cover used is from the Global Land Cover Classification (GLCC) raster created by the USGS ( http://edc2.usgs.gov/glcc/glcc.php). In Niger, land cover classifications included deciduous woodland/shrub land, grassland, croplands and desert (sandy, stony and bare rock). Walking speed across the different land cover types is summarized in Table 2.

#### Water bodies and Streams

Data on the location of streams and water bodies were taken from the Digital Chart of the World (DCW, 1990). The water body dataset was rasterized and merged with the land cover classification dataset mentioned previously to provide data on barriers of access during the rainy season.

#### Elevation and Slope

Digital elevation for Niger was obtained from the USGS (GTOPO30 ( http://www1.gsi.go.jp/geowww/globalmap-gsi/gtopo30/gtopo30.html)) at a 1 km resolution. A percent slope surface was created in ArcGIS 10 using the slope command in the spatial analysis toolset. Once created, the slope surface was reclassified to the values in Table 3. Although much of Niger is flat there are locations near Agadez and the desert that contain steep slopes. In these locations travel speeds were adjusted based on the values in Table 3.

### Analysis

No data are available about health facility service regions in Niger nor are there data about services at facilities from which estimates could be made about situations in which individuals might travel farther for better services. Thus, for the purpose of this study, settlements were assumed to use the nearest health facility, even though alternative choices may sometimes be made [4].

(i) **Accessibility of health facilities in Niger.** Travel times to all health facilities were calculated for the wet and dry season using two modes of transportation – foot and local vehicle (i.e. bus or ‘bush taxi’). Four friction surfaces were calculated using the accessibility model described earlier. Essentially one surface describes the walking travel speed during the dry season (walkdry), a second describes the walking travel speed (with impediments due to flooding) during the wet season (walkwet), a third describes vehicle travel speed along roads and walking speed for all other surface types during the dry season (vehicledry) and lastly the fourth surface describes vehicle travel speed along roads and walking speed for all other surface types (with impediments due to flooding) during the wet season (vehiclewet). For the latter two surfaces, walking speed has been included to account for people that must combine walking with vehicular travel.

(ii) I**dentifying critical populations at risk**. Travel times were extracted from each of the four outputs to the settlements data and summarized using seven time intervals that include (< 1 h, 1–2 h, 2–4 h, 4–12 h, 12–24 h, 1–2 days, >2 days). Previous studies have used a 1-hour [4,77] to health service criteria when investigating access. More recently, [5] found that hospitalized malaria incidence more than doubled when travel time to the nearest health care facility took longer than 2-hours and mortality doubled after a 4-hour walk [6]. Although, we will use the intervals described above to determine how accessible health facilities are for the people in Niger, for the purpose of this study settlements greater than 4-hours walk from a health facility were considered to be inadequate. Populations with the least access were calculated for each of the four accessibility outputs.

(iii) **Influence of distance on vaccination rates.** We further examine the impact of distance from a health center on the vaccination status of children aged 12–59 months. In Niger, health centers are important in the supply chain of vaccines [78], provision and co-ordination of vaccines on-site as well as off-site (through outreach and mobiles services to villages) [79]. Niger’s Ministry of Health aims to provide a “Minimum Activity Package,” (i.e. a minimum set of health services to people living within 5 km (approximately 1-hour walking speed) of a health center) [79]. Outside of this radius, populations are served through outreach and mobile services, thus making access to health centers from villages as well as access of villages by health centers important factors in coverage rates.

Demographic and Health Survey (DHS) completed before the rainy season in 1998 was used. The DHS data collection procedures are approved by the ICF Institutional Review Board, Calverton, MD, as well as by a review panel in Niger that approves research studies on human subjects. The analyses in this study represent secondary analyses of de-identified DHS data and we did not seek additional approval from an Institutional Review Board. Cluster locations are randomly scrambled to maintain anonymity, therefore we used the suggested methods of categorization [80] and grouped the data into dichotomous variables. Using the cost-surface-derived time measures, the number and proportion of children living in locations were categorized within and beyond 1-hour (walking) of a health center (a criteria that has been used previously [4,77]) and stratified into rural and urban.

Next we related the DHS, which included geocodes for clusters, to our database of health centers. We used census enumeration areas, generally corresponding to a rural village or an urban city block [81]. The outcome variable—“complete vaccination”—was defined as receipt of eight vaccine doses—Bacille Calmette-Guerin (BCG), 3 doses each of Diphtheria Pertussis Tetanus (DPT) and Oral Polio Vaccine (OPV), and measles—by 12-months of age. We did not examine the timeliness of *each* vaccine dose, but rather the completion of the vaccination schedule by 12-months of age.

Lack of vaccination was gauged from the health card or by mother’s recall. Maternal recall of vaccination status has been shown to be accurate [82], increasing our confidence in using maternal recall as a way to gauge lack of vaccination. Constraining the sample to children aged at least 12-months allows a period of three months for children to receive the measles vaccine, which is recommended to be received at 9-months of age in Niger [83]. For vaccinated children, vaccination by 12-months of age was determined by comparing the immunization date on the health card (a vaccination record filled out by a health worker) and the birth date.

Cluster-level independent variables included the type of location (urban/rural) and whether the cluster was within 1-hour from a health center (1; 0 otherwise); household-level variables included maternal (1 if mother attended any school, 0 if not) and partner education (1 if partner attended any school, 0 if not), whether the delivery of the child was assisted by a nurse/midwife (yes coded as 1, no coded 0), and household resources (see below).

The DHS household schedule collected information on whether the house had electricity, drinking water access (source of drinking water and time to source), quality of flooring used in the dwelling, and sanitation (type and whether it is shared between more than one household). It also included data on assets owned by the household (radio, TV, fridge, bike, motorbike, and car), providing data for a “household resources” index based on the methods of Alkire and Santos [84]. If the household had a car or had more than one of the other five assets above, it received a score of 1; if it had one or fewer of the five other assets and did not own a car, the household received a score of 0. If the household’s access to drinking water lay less than 30 min away and if it had access to an improved source of drinking water as defined in the Millennium Development Goals (MDG) [85] the household received a score of 1; else 0. If the household had a flush toilet or a ventilated, improved toilet and did not share a toilet with other households, it received a sanitation score of 1. If, on the other hand, it had a traditional or no toilet facility or shared a toilet with other households, it received a sanitation score of 0. Electricity was coded 1 if the household had access; zero otherwise. The household received a score of zero for a dung, earth, or sand floor; 1 for an improved floor (ceramic tiles, cement, vinyl, or carpet). The five scores (assets, water, sanitation, electricity, and flooring) were aggregated to produce an index (ranging from 0 to 5) of resources available to the household.

Stata 11 was used for bivariate analyses and data management. Because (as reported below) distance to a health center was found to have opposite relationship to vaccine uptake in urban and rural clusters, we stratified the dataset into urban and rural clusters to examine the relationship between complete vaccine uptake and independent variables and conducted a multilevel analysis on only rural clusters. HLM 7 [86]. Our two-level model accounted for the hierarchical nature of the data due to two-stage sampling in the DHS and allowed us to estimate the impact of living less than 1-hour from a health center on complete vaccine uptake by 1-year of age while controlling for household-level variables.

$$\;\begin{array}{cc} \text{logit}\left({\text{p}}_{timely\;vaccination}\right) & =\text{log}(\text{p}/\left[1-\text{p}\right])\\ & =\beta_{0}+\beta_{1}{\text{H}}_{\text{ij}}+\beta_{2}{\text{C}}_{\text{ij}}+{µ}_{\text{j}} \end{array}$$

where μ_j_ ~ N(0, $\tau_{0}^{2}\tau_{0}^{2}$), p is the probability of timely vaccination, H represents household-level variables, and C represents cluster-level variables for individual i in cluster j. The assumption is that the outcome, Y_ij_ has a Bernoulli distribution with a variance of p(1-p). Maximum likelihood estimation was used in HLM 7.

#### Model building

Starting with a “null” model, we sequentially built a multilevel model using HLM 7. Noting the deviance in each model, we tested for a significant change in deviance with a chi-square test. We first added the level-2 variable (time to a health center <1-hour) into the model and then examined whether this variable remained a significant predictor after including household-level factors at level-1. We included partner’s and mother’s education, mother’s access to birth assistance from a nurse-midwife, and household resources at level-1. Each level-1 variable was tested for random variation across clusters. Because there was no random variation across clusters, the cluster-level variable was included only in the intercept equation.

(iv) **Improving access through the placement of additional health facilities.** Outputs from (ii) were used to identify areas where access to health facilities was poor. To do this, areas where accessibility took longer than 4-hours were extracted for each of the four outputs (walkdry, walkwet, vehicledry, vehiclewet) from the settlements dataset. Population density estimates were created using the Kernel Density Estimate in ArcGIS10 Spatial Analyst to highlight the locations of highest population. The purpose of this analysis is to investigate how accessibility may be improved through the placement of additional health facilities in areas where their impact will be greatest (i.e. highest populations that have the least access). Through the creation of hexagons at 20 km (utilizing a Create Hexagons Tool [87]) (representing maximum 4-hour walking access), a grid was created that was overlaid on areas where population without access was greatest during the wet and dry season. Population totals were calculated for each hexagon and the highest populated cells were selected to place the health facility. Placement of health facilities occurred in cells with populations greater than 20,000 people for 20 km hexagons. Each health facility placement within a hexagon was constrained to be near a road to allow delivery of medical supplies, be placed in an existing settlement and located near to the location of the population median for that cell. Once the new facilities had been added a new cost distance surface was created (as described earlier) and travel times to health facilities extracted for each settlement and reanalyzed.

## Results

Here we present our findings starting with an assessment of the current distribution of population and health facilities in Niger. Next we used GIS to analyze how accessible health facilities are during the dry and wet season, identify areas with inadequate access to health resources and determine whether utilization of clinics (based on vaccination rates) was influenced by distance. Finally, we evaluated how access to health services may be improved by examining the potential impact that adding health facilities could have if they were located to optimize access to those currently least well served.

### Population and infrastructure in Niger

**Figure 1:**
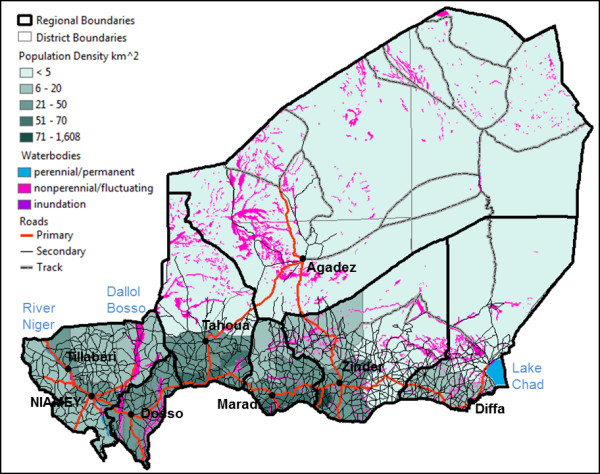
**Geography of Niger.** Map illustrating distribution of people and connectivity between departments. Population density by department is used to illustrate distribution of the population. Four road types are shown on the map and include Primary (paved), Secondary and Tertiary (non-paved, composed of sand and gravel), and Track (mainly through sandy desert). Permanent and fluctuating water sources are highlighted.

The majority of the population in Niger lives in the southern part of the country along the Nigerian border (Figure 1). Population densities are highest in the cities of Niamey, Agadez, Zinder, and Maradi with greater than 1200 people/km^2^. Much of the northern region of Niger is covered by the desert with a population density of less than 5 people per square kilometer (Additional file 1: Table S1). Distribution of roads, similar to population, is also heavily concentrated in southern Niger. Niger contains 63,000 km of road, ten percent of which are paved and link the main cities (Figure 1). Niger is comprised of 36 districts, six of which do not have any health facilities located within their boundaries (Additional file 1: Table S1,Additional file 2: Figure S1). Eleven districts are without a hospital or maternity center (Additional file 1: Table S1). Ratios of people per health facility served ranged from 208,000 to 7000 (Additional file 1: Table S1).

### How accessible are the current health facilities?

**Figure 2:**
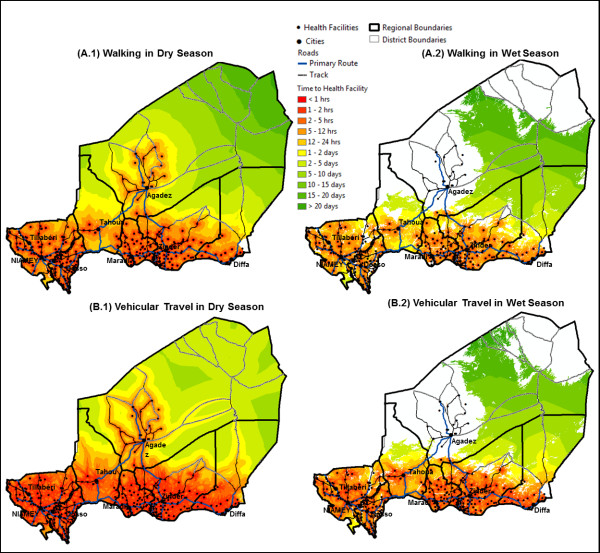
**Change in accessibility to health facilities during the wet and dry season by foot and vehicular travel. A** illustrates how accessible the health centers are when walking during the (A.1) dry season and (A.2) wet season. **B** illustrates how accessible the health centers are when using vehicular travel during the (B.1) dry season and (B.2) wet season.

Accessibility to all health facilities during both the dry and wet season is shown in Figures 2A and 2B. During the dry season, accessibility using vehicular travel provided faster access to all health facilities than by foot for much of the South of Niger, with exception in Tahoua where there was a lack of health facilities. The most inaccessible regions were east of Agadez and Zinder, where few paved roads exist across the Tenere Desert (Figure 2A.1 and 2B.1). Although this region is highly inaccessible, accessibility can be reduced substantially when travelling by local bus or cars (bush taxi’s) to within 2–4 days vs. greater than 10 days by foot/camel.

During the dry season 39% of the population was within 1-hours walk of a health facility, which was reduced to 24% during the wet season (Table 4; Figure 2A.2). Forty-three percent of the population was within 1-hour of a health facility when using vehicular travel (e.g. bus or “bush taxi”) on roads (Table 4; Figure 2B.1). For vehicular travel, accessibility was reduced to 26% during the wet season and is clearly seen in the size of the white areas on the wet season maps in Figures 2B.1 versus 2B.2. Furthermore, accessibility to 84 health centers may be affected during the wet season by inundation and/or fluctuating water levels in non-perennial rivers as illustrated in Figure 2B.2. These include health facilities located along the River Niger and its tributaries, in the Dallol Bosso river valley and in the region of Agadez.

**Table 4:** Percentage of the population with walking and vehicular access to health facilities during the dry and wet season

	**Dry season**		**Wet season**	
**No hours**	**% total population walking**	**% total population vehicle**	**% total population walking**	**% total population vehicle**
**<1**	39.11	42.90	24.09	26.34
**1–2**	13.93	24.37	5.64	10.36
**2–4**	18.17	22.79	13.23	21.05
**4–12**	18.08	7.72	23.24	28.50
**12–24**	6.96	0.23	9.66	3.63
**1–2 days**	0.85	0.92	5.80	0.14
**>2 days**	0.48	0.13	0.33	0.20
**Inaccessible/ No data**	2.43	0.94	18.01	9.78

Number of hours it takes to get from a clinic was estimated using two modes of travel, walking and local vehicles. Travel times across different land types and road types were based on travel speeds in Table 2– 4.

**Figure 3:**
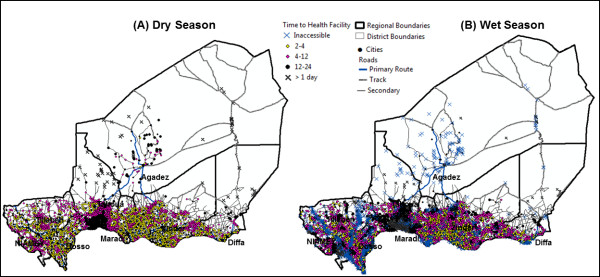
**Spatial distribution of settlements and accessibility to health facilities.** The maps show the spatial distribution of settlements that are greater than 4 h walk (pink dots) and 12 h walk (black dots and crosses) from a health facility during the dry and wet season.

The figures illustrate that accessibility can clearly change between seasons and that the changes impact a large proportion of the population (Figure 2 and Figure 3), by restricting the availability of health facilities. By combining the accessibility surfaces with the settlements data we were able to determine populations with the least access. Figure 3A and 3B show the number of hours required by each settlement to walk to a health facility during the dry and wet season. A distinct cluster was identified in the southern part of Tahoua where access to a health center was found to take in excess of 12-hours during both the dry and wet season (black dots, crosses) (Figure 3A vs. 3B), mainly due to the lack of health facilities in this area (Additional file 1: Table S1). In Tahoua, settlements that were between 4–12 hours walk from a health facility during the dry season take even longer to reach during the wet season (12–24 h). Several settlements that were accessible became inaccessible (blue crosses) during the wet season (Figure 3B). This is clearly visible in Agadez and along several tributaries of the River Niger.

### Impact of distance from health facilities on vaccination rates

Among 264 clusters with children aged 12–59 months, 177 were rural and 87 were urban clusters. Overall, 114 clusters (43%) representing 39% of 2429 children (children who either lacked vaccination or for whom the dates of vaccination could be determined) were located within 1-hour (walk time) of a health center. However, only 21% of rural clusters (24% of rural children), compared to 76% of urban clusters (86% of urban children) were within 1-hour walk time of a health center.

Among urban children, 50% were completely vaccinated by 12-months of age compared to only 8% of rural children. In bivariate analyses using urban clusters, somewhat surprisingly, 67% of children living greater than 1-hour from a health center compared to 44% of those living within 1-hour from a health center were completely vaccinated by 1-year of age (p = 0.001). In rural clusters, however, 11% of children living within 1-hour from a health center were completely vaccinated compared to 7% of children living greater than 1-hour from a health center (p = 0.005).

Table 5 presents results from the most parsimonious models explaining complete vaccination by 12-months of age in rural clusters. Living within 1-hour of a health center was related to 1.88 (95% C.I. = 1.00-3.54) times higher odds of complete vaccination by 12-months of age compared to children who lived greater than 1-hour from a health center (p < 0.05). After controlling for household level factors, children living in clusters less than 1-hour from a health center had 1.81 (95% C.I. = 0.99-3.29) times higher odds of complete vaccination compared to children who lived greater than 1-hour from a health center (p < 0.10) (Table 5). We did not include partner’s and mother’s education in the final model in Table 5 due to lack of variation in these variables in rural Niger (only 7 and 8% of mothers and partners were educated respectively); these two variables did not result in a significant decrease in deviance using a chi-square test of significance. Compared to a null model, models 1 and 2 in Table 5 each resulted in significantly lower deviance (p < 0.05).

**Table 5:** Impact of living within 1-hour walking time from a health center in rural Niger

**Model**	**1**	**2**
	**Walking time only**	**Walking time and household level**
*Cluster-level Variable*		
Within 1-hour walking time	1.88 (1.00-3.54)**	1.81 (0.99-3.29)*
***Household-level Variables***		
Delivery assisted by nurse-midwife (NM)		4.46 (2.43-8.17)***
Resources available in household		1.55 (1.07-2.23)**
**Random Intercept**	0.05 (0.04 - 0.08)***	0.04 (0.03-0.06)***
**Deviance (−2 log-likelihood)**	4313.86	4272.67
**Individuals**	1852	1852
**Clusters**	177	177

*p < 0.10, **p < 0.05, ***p < 0.001.

### Potential placement of additional health facilities

Not only can distance impact vaccine uptake rates, but also increase likelihood of hospitalization and mortality. For example, mortality in under 5-year olds has been shown to double when access is greater than 4-hours walk [6]. Therefore improving health-care access can help increase survival in sub-Saharan Africa, as demonstrated by Schoeps *et al.*[6]. This can be accomplished by reducing the travel time to health facilities by the placement of additional health facilities in areas where populations have the least access.

**Figure 4:**
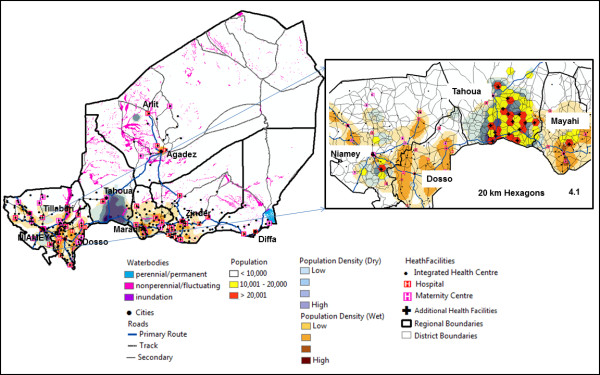
**Proposed placement of new health facilities in population densities with the least access to a health facility.** The maps show the most likely placement of proposed new health facilities within the densely populated regions using two different widths of hexagon. Proposed new health facilities were placed in the red hexagons (4.1).

We used the data we assembled and GIS-based analytical methods to investigate the potential impact of placing additional health centers in areas that had the least access. First, we determined which settlements were more than 4-hours from health facilities during both the dry and wet season and identified areas with the highest population density as shown in Figure 4. Several overlapping areas were found in Tillaberi and Maradi with the largest occurring in Tahoua. In the uranium mining area near Arlit a highly inaccessible area was found. This was excluded since the mining company provides health care for employees [88].

We then assessed how access to health services can be improved by examining the potential impact that additional facilities could have if they were located in currently underserved regions. Population was calculated for each 20 km (representing maximum 4-hour walking access) hexagon and seventeen health facilities were placed within hexagons of highest population (> 20,000 people) (Figure 4.1). Adding these facilities would improve health facility access to within 4-hours walk for approximately 730,000 people, an increase from 71% to 81% during the dry season (Table 4 vs. 6). During the wet season access would be improved for approximately 260,000 people, an increase from 43% to 46%. Accessibility by vehicular travel would also be greatly improved, most notably during the wet season. This would increase from 90% to 95% during the dry season and 57% to 72% during the wet season.

**Table 6:** Summary of improved accessibility through the placement of an additional 17 health centers in areas of most need

	**Dry season**		**Wet season**	
**No hours**	**% total population walking**	**% total population vehicle**	**% total population walking**	**% total population vehicle**
**<1**	43.59	76.26	25.63	41.16
**1–2**	16.22	13.41	6.24	12.24
**2–4**	21.58	5.72	14.79	19.28
**4–12**	14.02	1.02	27.79	15.79
**12–24**	0.82	0.18	6.30	1.19
**1–2 days**	0.81	0.91	0.80	0.11
**>2 days**	0.44	0.13	0.34	0.17
**Inaccessible/ No data**	2.51	2.36	18.10	10.05

## Discussion

This study highlights critical areas in Niger where health services/facilities may need to be improved using realistic travel time estimates to represent access times during the dry and wet season. The findings from this study highlight accessibility problems that are similar to those faced by many countries during the dry season (e.g. Kenya [4]; Yemen [2]) and wet season (e.g. Malawi [39]; Ghana [38] ). Several previous studies have used a 1-hour [4,77] to health service criteria when investigating access. If we compare our findings using this criterion then we can clearly see that in Niger, access to health facilities is highly inadequate with greater than 75% of the population having to travel more than an hour by foot to the nearest health facility during the wet season and greater than 60% of the population during the dry season. Access can be reduced slightly using vehicular travel (i.e. 73% and 57% of population is greater than 1-hour away from a health facility during the wet and dry season, respectively). As efforts increase to meet reduction in childhood mortality [89] it is clear that improving access and delivery of health care to reduce hospitalization (< 2-hour walk; current access is 29% (wet season) and 53% (dry season)) and mortality (< 4-hour walk; current access is 42% (wet season) and 71% (dry season)) are greatly needed. Vaccinations are an important preventive intervention for children for reducing mortality and incidence of disease [90-92], therefore improving access to health facilities that can provide vaccinations is essential.

In this study, we found that children living in rural clusters within 1-hour of a health center had higher odds of being completely vaccinated by age 1-year compared to children living farther away, suggesting that residents within this travel time from a health center had better access to vaccination services than residents outside this travel time; this is in spite of outreach and mobile services having been developed to support the outlying population [79]. In urban areas, however, residents outside 1-hour from a health center had higher rates of complete vaccination, suggesting that the outreach strategy may function even more effectively than the stationary strategy in urban Niger [79]. In rural areas, our analysis suggests that the outreach strategy may have some room for improvement so that disparities between those living within 1-hour and those living farther away from health centers can be removed, This may be particularly the case where nomadic, seasonal movement is prevalent. Accounting for these migration patterns is difficult in Niger because of the paucity of data (see [93]) however, these kind of movements would be more likely to result in an underestimation of vaccination uptake during the wet season as nomadic groups move away from health facilities. Health Systems Strengthening funding was availed by Niger in 2009 to improve outreach and mobile vaccination services to people living farther from health centers [94]. However, it is conceivable that even mobile teams may find it difficult to reach villages farther away during the rainy season due to road deterioration making the choice of location for permanent and temporary health centers in rural Niger important. It is worth noting here that DHS cluster locations are randomly displaced to preserve anonymity, limiting our ability to identify specific villages farther away from health centers.

Although, we did not have detailed information on utilization of health facilities, as used in several studies [6,38-40] that looked at seasonal variations in health-seeking behavior, we were able to determine how physical access may change between seasons and illustrate populations with low access based on travel speed. Data on childhood vaccine uptake are from 1998. Though Niger had a DHS survey conducted in 2006, geocodes for clusters in that sample are unavailable [95]. Rates of complete vaccination by 1-year of age of children aged 12–59 months changed little between these two time points, however (13.1% in 1998 and 12% in 2006) increasing our confidence in the relevance of analysis based upon the 1998 data.

Overall, the methods applied in this study can be used to assess placement of facilities based on population and realistic travel times, key considerations suggested by Al-Taiar *et al*. [2] and Noor *et al.*[4]. We showed that we were able to use supplementary information obtained from a variety of sources such as news articles and travel blogs [37,59,60] to determine realistic travel times. In the future, it would be very useful to validate these ancillary data sources to ensure information provided on travel blogs are representative of local travel. The methodologies utilized here are useable in other locations where detailed data may be lacking. Mapped outputs from this study can be further integrated with other types of data such as mortality rates of births and treatable diseases such as polio, malaria and meningitis (see [96]), to identify critical areas where improvements to health services would be beneficial. The approach is also applicable for crisis management to identify the best placement of temporary/mobile facilities in regions where disease may be present during seasonal outbreaks (i.e. cholera, mosquito-borne diseases such as malaria during the wet season and pneumonia, meningitis and measles during the dry season as well as feeding centers during severe droughts) or to plan relocation of populations during disaster events such as illustrated in Haiti during the earthquake of 2010 [97]; or to identify placement of additional health centers along key migration routes as illustrated in Tomaszewski *et al.*[93] and Gele *et al.*, [40].

Changes in accessibility during the wet season are based on the assumption that flooding will occur yearly, which may not necessarily be the case, particularly during drought years. Therefore, the wet season estimates may present a worse-case scenario. However, between 1970 and 2000, Tarhule [23], found that 79 severe rainfall and flood events took place in Niger, indicating that flooding is not uncommon in this region. More recent news reports during 2009–2011 further highlight the extensive damage to infrastructure that can result in Niger as a result of flooding [24-26,73-76]. Thus, it is likely that during the wet season accessibility will be affected and therefore should be considered in the placement of health facilities.

In this study we used the location of health facilities for hospitals, maternity and integrated health centers. Although additional facilities may exist in Niger, no public information is available for these and was therefore not included however, when these do become available (e.g. location of health post), the inclusion of these locations would refine and improve the results presented here. Thus, for this study we assume that the closest health facility is used. This may not always be the case [4] since people may prefer to go to health centers where services are more reliable and facilities better equipped [98], therefore improvements to the analytical methods used here can be made in the future by including attributes of health facilities in the analysis (i.e. services provided, medicines available, capacity, and cost of services). By including limitations of some facilities in the analysis rather than assuming all facilities are accessed equally, it is likely that we would find an even larger proportion of people with inadequate access. Furthermore, we are aware that *physical* access to health care is only one component of access to health care. Factors such as perceived quality of health care services provided at public health facilities, trust in the health care providers, quality of and sensitivity in communication by care providers with the public, and ability to pay for care [99,100] as well as costs associated with time taken from income producing activities [101] are potentially all determinants of the complete package of access to health care that we do not address in this study.

Although we used realistic travel times in our analysis, further adjustments may be necessary. For example, walking speed is likely to be slower for sick adults [102] and adults carrying children [103] than for healthy adults walking on their own. Therefore it would be useful to validate these travel times in the future. In addition, it would be beneficial to include travel costs, as used in the study by Ewing *et al.*[39], to identify areas where costs may act as a further barrier to health-seeking behavior.

## Conclusion

This study found that there is substantial geographic variation in accessibility to health facilities in Niger and that the differences are not simply the obvious ones between the sparsely populated northern desert region that contains few roads and the rest of the country. Variation exists in the populated south as well. Results highlight critical areas in Niger where health services/facilities are lacking. In addition, analysis determined that the population served by health facilities will be severely overestimated if assessment of accessibility is conducted without consideration of seasonal differences in travel impediments. Numbers of people who are estimated to have limited or no access to health facilities increases substantially in the wet season and clusters of people in the south with limited access grow in size. Thus, in regions where precipitation may impede physical access, effects of seasonality should be factored in the distribution of health services.

## Competing interests

The authors declare that they have no competing interests.

## Authors' contributions

JIB conceived the paper. JIB, SK and WL performed the analysis; AMM advised on methods. JIB, SK wrote the paper with input from AMM. All authors read and approved the final manuscript.

## Supplementary Material

Additional file 1 **Table S1.** Distribution of health facilities and population by district in Niger. The table highlights the total number of hospitals, maternity and integrated health facilities located within each district (see Additional file 2: Figure S1). Average distance (km) between health facilities (+/− SE) is summarized by district. Districts lacking health facilities are illustrated. Click here for file

Additional file 2 **Figure S1.** Map illustrating district and regional level administration boundaries. Click here for file
