# Supplementary material for: It’s a long, long walk: accessibility to hospitals, maternity and integrated health centers in Niger
Source: Int J Health Geogr. 2012 Jun 27;11:24. doi: 10.1186/1476-072X-11-24 (PMC3515413; doi:10.1186/1476-072X-11-24)
Supplement: Additional file 1 — Table S1. Distribution of health facilities and population by district in Niger. The table highlights the total number of hospitals, maternity and integrated health facilities located within each district (see Additional file 2: Figure S1). Average distance (km) between health facilities (+/− SE) is summarized by district. Districts lacking health facilities are illustrated. [file 1476-072X-11-24-S1.docx]

**Table S1**. **Distribution of health facilities and population by district in Niger**. The table highlights the total number of hospitals, maternity and integrated health facilities located within each district. Average distance (km) between health facilities (+/- SE) is summarized by district. Districts lacking health facilities are illustrated.

| Region | District | Hospital (km) | Integrated Health Centre(km) | Maternity  (km) | Total Population | Population served by health facility | Area District (km^2^) |
| --- | --- | --- | --- | --- | --- | --- | --- |
| Agadez | Arlit | 119 (37.52)  (1) | 107(56.02)  (7) | 111(44.6)  (2) | 68,064 | 6,806 | 201,153 |
|  | Bilma | None | None | None | 8,458 | None | 277,591 |
|  | CU Agadez | 1.04  (1) | 1.04  (10) | None | 49,361 | 4,487 | 65 |
|  | Tchighozerine | 103(59.03)  (2) | 133(76.22)  (12) | 116(63.16)  (2) | 80,137 | 5,724 | 144,714 |
| Diffa | CU Diffa | 34(22.76)  (2) | 51(30.43)  (9) | 76(36.37)  (1) | 76,266 | 6,355 | 6,834 |
|  | Maine-Soroa | 76(38.59)  (1) | 60(33.94)  (7) | 76(38.59)  (1) | 82,102 | 9,122 | 15,441 |
|  | N'Guigmi | None | 105(79.79)  (3) | None | 28,229 | 9,409 | 123,724 |
| Dosso | Birni N'Gaoure | 52(35.34)  (3) | 50(33.16)  (9) | 34(19.4)  (1) | 202,301 | 15,561 | 4,498 |
|  | CU Dosso | 41(22.19)  (2) | 57(33.74)  (13) | None | 232,920 | 15,528 | 7,999 |
|  | Dogon-Doutchi | 61(34.75)  (3) | 66(42.12)  (17) | 63(40.16)  (5) | 305,377 | 12,215 | 11,092 |
|  | Gaya | 59(30.62)  (1) | 48(28.29)  (15) | 59(30.62)  (1) | 161,130 | 9,478 | 4,042 |
|  | Loga | 31(15.33)  (2) | 32(18.53)  (1) | 26.64(16.42)  (1) | 99,085 | 24,771 | 3,827 |
| Maradi | Aguie | None | 29(5.18)  (7) | 27(13.38)  (1) | 178,622 | 22,328 | 2,853 |
|  | CU Maradi | 4(1.8)  (2) | 4(1.8)  (8) | 4(1.8)  (2) | 113,657 | 9,471 | 70 |
|  | Dakoro | 56(26.07)  (1) | 63(32.31)  (12) | 56(26.07) (1) | 255,371 | 18,240 | 16,544 |
|  | Guidan-Roumdji | 36(20.16)  (1) | 39(23.16)  (9) | 36(20.16)  (1) | 214,193 | 19,472 | 4,762 |
|  | Madarounfa | 21(9.2)  (1) | 25(12.52) (13) | 21(9.2)  (1) | 194,571 | 12,971 | 3,588 |
|  | Mayahi | 35(17.67)  (1) | 42(21.83) (8) | 35(17.67)  (1) | 228,199 | 22,820 | 6,495 |
|  | Tessaoua | 40(20.26)  (1) | 47(26.96)  (9) | 40(20.26)  (1) | 204,522 | 18,592 | 5,104 |
| Niamey | CU Niamey | 7.5(3.81)  (6) | 8.3(4.2)  (34) | 8.4(3.97)  (7) | 397,766 | 8,463 | 293 |
| Tahoua | Abalak | None | None | None | 40,284 | None | 12,567 |
|  | Birni N'Konni | None | None | None | 253,487 | None | 4,836 |
|  | Bouza | None | 30(18.05)  (2) | None | 182,167 | 91,083 | 3,546 |
|  | Illela | None | None | None | 174,191 | None | 6,480 |
|  | Keita | None | None | None | 118,452 | None | 4,955 |
|  | Madaoua | None | 29(14.45)  (1) | None | 208,120 | 208,120 | 4,509 |
|  | Tahoua | 40(18.91)  (2) | 46(23.45) (13) | 39(20.32)  (3) | 229,117 | 12,728 | 9,122 |
|  | Tchin-Tabarade | None | None | None | 53,785 | None | 60,302 |
| Tillabery/ Tillaberi | Filingue | 63(29.41)  (2) | 71(42.72)  (17) | 63(40.49)  (4) | 286,642 | 12,462 | 24,153 |
|  | Kollo | None | 58.5(33.46) (19) | 87.1(31.6)  (1) | 203,068 | 10,153 | 7,970 |
|  | Ouallam | 54(35.93)  (1) | 69(35.74) (12) | 64(33.06)  (3) | 184,369 | 11,523 | 20,724 |
|  | Say | 65(29)  (1) | 70(36.27) (6) | 50(27.65)  (1) | 198,201 | 24,775 | 14,794 |
|  | Tera | 51(23.46)  (1) | 70(41.56) (17) | 59(34.48)  (2) | 284,137 | 14,206 | 14,960 |
|  | Tillaberi | 40(22.68)  (1) | 56.5(36.9) (19) | 52.5(34.69)  (3) | 164,220 | 7,140 | 8,156 |
| Zinder | CU Zinder | 0.66(0)  (2) | 0.66(0)  (9) | 0.66(0)  (2) | 119,838 | 9,218 | 97 |
|  | Goure | 62.4(35.19) (2) | 83.5(46.5) (14) | 61(26.06)  (1) | 160,130 | 9,419 | 89,234 |
|  | Magaria | 53.5(29.87)  (2) | 53.6(29.61) (16) | 45.5(25.35)  (1) | 347,686 | 18,299 | 8,011 |
|  | Matamey | 25.6(12.96)  (3) | 25.4(12.33)  (9) | 21.5(10.11)  (2) | 166,903 | 11,921 | 2,215 |
|  | Miria | 40.5(20.69)  (1) | 58.8(31.88)  (24) | 40.5(20.69)  (1) | 427,232 | 16,432 | 13,321 |
|  | Tanout | 66.8(31.5)  (1) | 58(30.61)  (9) | 66.8(31.5)  (1) | 188,098 | 17,099 | 33,064 |
