# Supplementary figures and images for: It’s a long, long walk: accessibility to hospitals, maternity and integrated health centers in Niger
Source: Int J Health Geogr. 2012 Jun 27;11:24. doi: 10.1186/1476-072X-11-24 (PMC3515413; doi:10.1186/1476-072X-11-24)

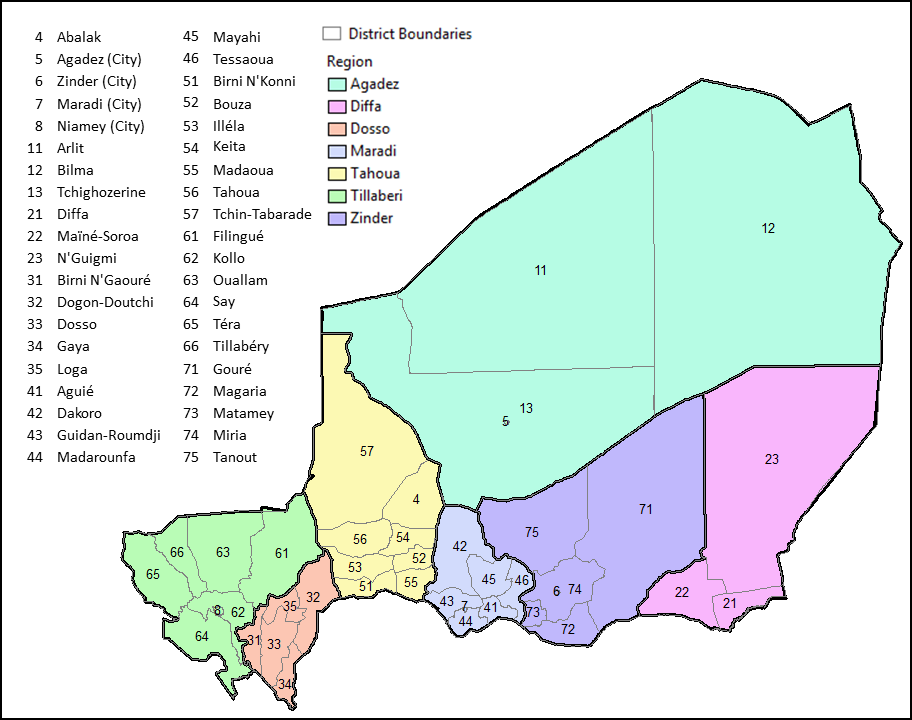

Supplement: Additional file 2 — Figure S1. Map illustrating district and regional level administration boundaries. [file 1476-072X-11-24-S2.png]
